# Developing a set of key principles for care planning within older adult care homes: study protocol for a modified Delphi survey

**DOI:** 10.1136/bmjopen-2024-090243

**Published:** 2025-01-28

**Authors:** Jonathan Taylor, Thais Caprioli, Jacqueline Damant, Yuri Hamashima, Sarah Jasim, Nick Smith, Madalina Toma

**Affiliations:** 1 Nuffield Department of Population Health, University of Oxford, Oxford, UK; 2 Department of Primary Care and Mental Health, University of Liverpool, Liverpool, UK; 3 Care Policy and Evaluation Centre, London School of Economics and Political Science, London, UK; 4 Population Health Sciences, University of Bristol, Bristol, UK; 5 Centre for Health Services Studies, University of Kent, Canterbury, UK

**Keywords:** Protocols & guidelines, Social Support, Nursing Homes, Person-Centered Care, Caregivers, Quality in health care

## Abstract

**Background:**

Older adult care homes in England are required to develop care plans on behalf of each of their residents and to make these documents available to those who provide care. However, there is a lack of formal agreement around the key principles that should inform the development of care plans in care homes for older adults. Using a modified Delphi survey, we intend to generate consensus on a set of key principles that should inform the care planning process.

**Methods and analysis:**

A two-stage modified Delphi survey will be used to try to reach a consensus on a set of key principles to inform care planning within older adult care homes in England. An interdisciplinary panel of approximately 50 people with experience in care planning will be convened and invited to provide feedback on a set of key principles. We will use an iterative, quasi-anonymous, multistage approach with controlled feedback. In the first round, panellists will be asked to provide feedback on a draft document whose contents have been informed by a systematic scoping review and consultations with care home staff. The first round will be administered and subsequently analysed. The results from the first round will be fed back to the panel members and panellists will be asked to complete a second survey. In each round, panel members will use a 5-point unipolar scale to rate their agreement with the item. Consensus will be considered if ≥75% of participants rate an item as 4–5.

**Ethics and dissemination:**

This study to which this protocol relates has been granted ethical approval by the University of Kent’s Division for the Study of Law, Society and Social Justice Research Committee Ethics Panel (reference: 1006) on 9 April 2024. The results of this project will be disseminated through conferences and one or more peer-reviewed journals. In a subsequent research phase, the research team plans to share the key principles document developed through this modified Delphi survey with care home residents and their families and friends. We plan to invite their feedback through a series of focus groups with a view to developing a related document for the family and friends of care home residents.

**STRENGTHS AND LIMITATIONS OF THIS STUDY**

- The Delphi method enables people with a range of professional experiences to anonymously share their knowledge and experience through a structured and iterative feedback process.
- The decision to collect participants’ feedback through an online survey will allow for faster data collection than an in-person survey.
- One of this study’s potential weaknesses is a large drop-off in the number of participants between the first and second surveys and so several steps will be undertaken to maximise the response rate.
- One of the study’s weaknesses is that the Delphi panel will not include care home residents or their families and friends; families and friends’ feedback will be collected as part of a future research project, with a view to developing a related resource for this group.
- Gaining consensus through a modified Delphi survey will not result in new evidence and so the resulting resource would benefit from being tested in a care home setting.

## Introduction

 An estimated 260 000 people aged over 65 live in older adult care homes in England.^1^ These homes are responsible for providing care and support while assisting their residents with daily activities such as eating, washing, dressing and socialising. To meet these needs, care homes must assess their residents’ needs and develop individual care plans. The health and social care services regulator in England, the Care Quality Commission (CQC), has defined care planning as a process ‘focused on the person’s whole life, including their goals, skills, abilities and how they prefer to manage their health’.^2^ Care homes are required by the CQC to ensure that the people they support are involved in the ‘planning, management and review of their care’. The CQC also stipulates that care providers must ensure that a resident’s care plan is available to all staff involved in the individual’s care.^3^

While care planning aims to understand a person’s present circumstances and preferences, most research has focused on advanced care planning (ACP).^4^,^6^ ACP, which should form part of the wider care planning process, relates to future care provision and is often focused on palliative care. ACP often takes place if it is anticipated that someone’s condition will deteriorate in the future.^7^ Much of this research has sought to examine the benefits of ACP for residents, families and healthcare systems.^8 9^

Researchers have also investigated specific care planning interventions. Studies have explored, for example, the efficacy of employing a biographical approach,^10^ integrating quality-of-life tools into care plan frameworks^11^ and implementing a case conference model in care planning.^12^ Most recently, qualitative research has found that care planning practices can vary considerably between care home settings.^13^

This study aims to establish consensus on a set of key principles that will inform how care planning is conducted by health and social care professionals in older adult care homes in England. The intended outcome is to create a document whose contents will be acceptable to care home practitioners and will help to ensure that care planning is consistently conducted in a person-centred way.

## Methods and analysis

### Justification for study design

A modified Delphi survey will be used to develop an information resource which describes a set of key principles relating to care planning in residential care settings for older adults.^14^ Delphi surveys have previously been used to develop best practice guidance, including guides related to the care of older adults.^15 16^ The modified Delphi technique will enable panellists to provide feedback that will be anonymous to all but the researchers. This approach minimises bias related to group conformity and the likelihood that a single individual will dominate the discussion.^17^

This study will further seek to minimise potential bias during both the recruitment and survey phases. Survey questions will be written in a neutral tone to reduce data collection bias. In order to reduce selection bias, a diverse range of organisations will be contacted to recruit participants, reducing the risk of over-representation from particular regions or professional backgrounds.^18^

Panel members recruited from across England will be invited to comment on a draft key principles document developed by the research steering group (RSG). The contents of this document will be turned into a series of statements each comprising a single sentence or bullet point. Panel members will be provided with a copy of the document as a PDF and will be invited to rate each of the statements through an online survey developed in Qualtrics.

The first draft of the key principles document was informed by three strands of work:

1. Consultations with people involved in providing care and support within care home setting—including activity coordinators, general practitioners, nurses, care home managers and deputy manager.
2. The findings of a systematic scoping review conducted during an earlier phase of this project.^6 13^
3. Ongoing input from two relatives of care home residents. These individuals assisted in developing the topic guide used as part of the consultations and the search strategy used as part of the systematic scoping review. As part of this modified Delphi study, these individuals were actively involved in drafting the first version of the key principles.

Consultations with people who provide care and support within care homes and were involved in care planning revealed a wide variety of approaches to care planning and limited evidence that care planning was being conducted in a person-centred way.^13^ Similarly, the scoping review identified inconsistencies in the interventions designed to promote care planning provided to staff and residents’ family and friends.^6^ This study builds on these findings by seeking to establish a consensus around a set of key principles to inform person-centred care planning in older adult care homes in England.

### Research steering group

The RSG (n=9) will include researchers from five academic institutions across England with backgrounds in care home research, and two patient and public involvement and engagement (PPIE) members who are experts with lived experience of a relative residing in a care home. The RSG will draft a key principles document and the modified Delphi survey, recruit panel members and circulate the content of the Delphi rounds. The RSG will not participate in the surveys; rather, they will supervise and monitor the process.

### Recruitment

Panellists will be recruited by purposive and convenience sampling techniques and will be approached through different methods. First, RSG members will use the CQC’s website to identify older adult care homes in their region and invite their staff members, via email, to take part in the study. Second, the study will be publicised by relevant intermediary organisations. These will include care home associations: local Enabling Research in Care Homes networks, the National Activity Providers Association (NAPA), the National Care Forum (NCF), the British Society of Gerontology’s Special Interest Group on Care Homes, trade unions and charities that advocate for care workers. Third, the RSG will be supported by PPI experts who will raise awareness of the study through their networks. Fourth, the RSG members will approach care homes that have taken part in previous research studies. These homes will include, for example, people who took part in the previous consultation work.^13^ This recruitment strategy, which will engage organisations across England and groups representing various sectors of the care home workforce, is designed to maximise the likelihood of assembling a multidisciplinary panel.

Finally, the RSG will contact key individuals from the following organisations to invite them to participate: CQC, NCF and the NAPA.

All prospective panellists will be made aware of the eligibility criteria, details of which are below. Prospective panellists will also be encouraged to share details about the study with people, such as those working at a different care home within the same chain, who would meet the eligibility criteria. We plan to begin recruitment of round 1 panel members in June 2024 and close data collection for the second round in October 2024.

### Eligibility criteria

To become a panel member, panellists must confirm that they are:

- Over the age of 18.
- Someone who has been involved in care planning in older adult care home settings in one or more of the following ways:
  - Writing care plans.
  - Reviewing the contents of care plans.
  - Using a care plan as part of providing care and support.
  - Supervising care planning.
  - Delivering training relating to care planning.
  - Contributing to one or more sections of a care plan.
  - Other (participant to provide more detail).

### Panel size

The panel size of Delphi studies varies widely, and no standardised size exists.^19^ Having reflected on several factors, including the purpose of the study, the complexity of the problem, the homogeneity of the sample and available resources, we aim to recruit a panel comprising a minimum of 50 panel members.^19 20^

### Anonymity

This project will be conducted quasi-anonymously. Panellists’ responses will be anonymous to one another but not to RSG members.^21^ Maintaining the anonymity of panellists is important to limit bias related to group conformity and/or dominance.^19^ Once the second round of the modified Delphi survey is completed, panellists who have participated in both rounds will be asked if they wish to remain anonymous or receive acknowledgement in the publication.

### Survey development

The survey questions directly relate to the information contained within the draft key principles document produced by the RSG. This document comprises seven sections, each presenting a series of statements. The statements will comprise a single sentence or bullet point contained within the key principles document.

The survey will be developed in Qualtrics. The round 1 survey will ask panellists to complete a 5-point unipolar scale question for each statement. A 5-point unipolar scale has been selected to meet the conflicting goals of offering enough choice to measure panellists’ strength of opinion while also ensuring that the items are easily understood by respondents.^22^ Respondents will be invited to rate each item as follows: ‘1=not at all important’, ‘2=slightly important’, ‘3=somewhat important’, ‘4=very important’, ‘5=extremely important’. The wording of these statements has been used in previous Delphi studies.^23 24^ A midpoint option might potentially be misused for an option when respondents are not familiar with the statements or when they feel the answer may depend on circumstances. We will, therefore, also include an ‘I don’t know’ option in addition to these five options.^25^

In the first of two rounds, panellists will also be invited to suggest revisions to the wording of the statements, suggest additional content, comment on the order of the statements and provide further comments. In both the first and second rounds the order of the sections, but not the statements, will be randomised to minimise the risk that panel members will invest more time reviewing early sections or become collectively biased due to previous responses.^26^

To pilot the modified Delphi survey and to avoid introducing bias when drafting the key principles, two people involved in providing care and support within care home settings, who meet the eligibility criteria that panellist are required to fulfil (set out above), will be asked to give feedback on the clarity and appropriateness of the survey questions we plan to use in the first round by completing a draft version of the survey hosted on Qualtrics (see onlinesupplemental files 1^2^). These respondents will not take part in the final modified Delphi surveys. The survey will be modified based on the feedback received.

### Definition of consensus

There is no agreed definition for what constitutes consensus within a Delphi study. Previous studies have defined a consensus as being between 51% and 80% agreement.^21^ This study conservatively defines consensus as being when ≥75% of panel members rate a statement as ‘4=very important’ or ‘5=extremely important’ on the 5-point unipolar scale. This threshold is consistent with previous research studies.^21^

### Enhancing response rate

Panellist fatigue is often associated with Delphi surveys.^17^ Several methods will be used to minimise attrition and improve response rates. The participant information sheet will include a paragraph explaining the importance of completing the Delphi process.^27^ Panellists will also be made aware that if they complete the survey online, they can submit their answers in more than one sitting. This step will be taken because we anticipate that people involved in providing care and support within care home settings, will have competing priorities and so may not have the time to complete the survey in a single sitting.

Offering alternative methods of data collection has been found to improve retention rates in longitudinal surveys.^28^ For this reason, panellists will also be given the option to complete the survey over the telephone or by emailing their responses to a member of the research team. If survey responses are provided over the telephone or by email, a member of the research team will input panellists’ answers into the online survey on their behalf. Prospective participants will also be made aware that they can request that a printed version of the key principles document be sent to them in the post.

Finally, we will minimise missing data by requiring panellists to answer all the questions. Participants will be free, however, to select ‘prefer not to say’ when answering questions about their demographic information and professional backgrounds.

Panellists will be provided with a £25 voucher for each survey that they complete. These sums reflect the National Institute for Health and Care Research’s recommended rates of reimbursement.^29^

### Efforts to minimise fraudulent survey responses

Research projects which offer financial reimbursements to online survey respondents can attract fraudulent responses that compromise the validity and interpretability of results.^30^,^32^

Several steps will be taken to minimise the inclusion of fraudulent data. Recruitment conducted via social media can make it easier for fraudulent respondents to take part in online surveys.^31^ For this reason, panel members will not be recruited through social media and will instead be contacted via emails to individual care homes and relevant intermediary organisations. Email recipients will be asked not to promote this research opportunity via social media.

To detect bots, the survey will include a Completely Automated Public Turing test to tell Computers and Humans Apart (CAPTCHA). Panel members who pass the CAPTCHA test will be asked to complete a series of questions to assess their eligibility. When developing the survey in Qualtrics the ‘Bot Detection’ option will be enabled. This makes it possible to track which responses are likely bots using reCAPTCHA V.3.^33^ Participants will be asked to type out ‘I am answering the screening questions honestly’. Only participants whose answers meet our eligibility criteria will be invited to complete the full survey.

Once panel members have completed the full survey, responses will be reviewed by at least two members of the RSG who will be attentive to the following issues: inconsistencies in the participant’s name and email address, the time taken to complete the full survey and many responses received within a short period. If the RSG reviewers believe a survey response is fraudulent, they will discuss this with the wider RSG before informing the participants of their decision. The participant information sheet will explain that the RSG reserves the right to withhold a voucher if they believe the response is fraudulent.

### First round

Round 1 panel members will be provided with (a) a copy of the draft key principles document, (b) a link to the online survey, (c) a participant information sheet and (d) a briefing document which will explain the process that led to the development of the draft key principles document. Participants will be asked to consult the key principles document when completing the survey questions. This approach is consistent with previous modified Delphi studies which have presented panellists with a set of prepared statements, developed through prior research activity, to establish consensus around a set of guidelines and preferred practices.^1534^,^36^

The draft document will comprise seven sections. Each section will present a series of statements. Round 1 panellists will be asked to complete a five-scale unipolar question for each statement. Panellists will also be asked to indicate how frequently they believe care plans should be reviewed. Panellists will be asked to respond to approximately 70 statements. Panellists will have the option to select ‘I don’t know’ for each statement. For each statement, panellists will also be invited to suggest revisions to the wording of the statements, the order of the statements, suggest additional content and provide further comments or questions. Panellists will also be provided with the opportunity to make any additional comments.

The round 1 survey will also include questions about panellists’ professional backgrounds, such as job titles and time spent working within the care home sector and demographic details. This information will provide a clearer understanding of the panel members’ characteristics.

Panel members will be asked to provide their email addresses so that they can take part in the second round. A reminder to complete the survey will be sent via email to everyone who has not completed the round 1 survey after 10 working days.

### Second round

To take part in the second round, panel members will have to have completed the first round. In round 2, panel members will be provided with (a) a copy of the revised key principles document, (b) a revised survey, (c) a copy of their round 1 response, (d) an anonymous summary of other panel members’ responses and (e) an explanation of the revisions that have been made. The explanations will be based on participants’ responses to the free text questions included in the first Delphi survey. This information will be presented in an anonymised form to reduce the risk of authority bias.^37^ This approach is consistent with previous modified Delphi studies.^38^ Participants will be asked to consult the revised key principles document along with the explanation of the changes when completing the second-round survey.

The revised survey will present a series of five-scale unipolar questions for each statement in the revised key principles document. Panellists will also have the option to select ‘I don’t know’ for each statement. The revised survey will not give respondents the chance to provide further qualitative feedback on the statements.

A reminder to complete the survey will be sent via email to everyone who has not completed the round 1 survey within 10 working days.

### Analysis of round 1 responses

Following the first round, the unipolar-scale scores will be summarised and presented as frequencies and mean ratings. The RSG will review all open-text responses to contextualise the quantitative responses. The views of all panellists will be given equal weight.

If at least 75% of panellists rate an item in the lower two categories (‘not at all important’, ‘slightly important’) or in the higher two categories (‘very important’, ‘extremely important’), we will consider consensus as having been reached and the item will be removed or retained, respectively. When calculating panellists’ responses, we will include those who select the ‘I don’t know’ option to avoid inadvertently inflating the number of statements exceeding the 75% threshold. If a majority of panellists select ‘I don’t know’ for a given item or across a majority of items, we will examine the potential reasons for this pattern (eg, a lack of clarity or relevance of the items). Based on this assessment, we will consult with the RSG to determine whether to revise the items or exclude them the second round. Items where ratings do not meet the consensus threshold will also be reviewed by the RSG, considering the qualitative responses received, and revised accordingly.

A thematic analysis of free-text responses will be undertaken in NVivo V.14. After coding a subset of responses, two researchers will meet to compare codes and agree on a coding framework that one researcher will subsequently apply to the remaining data. The RSG will develop a revised key principles document based on the results of the first-round analysis.

### Analysis of round 2 responses

Following the second round, the unipolar-scale scores will be summarised and presented as frequencies and mean ratings. The views of all panellists will be given equal weight.

If at least 75% of panellists rate an item in the lower two categories (‘not at all important’, ‘slightly important’) or in the higher two categories (‘very important’, ‘extremely important’), we will consider consensus as having been reached and the item will be removed or retained, respectively. As with round 1, when calculating panellists’ responses, we will include those who select the ‘I don’t know’ option to avoid inadvertently inflating the number of statements exceeding the 75% threshold. The RSG will develop a final key principles document based on the results of the second-round analysis.

### Public involvement

Two relatives of care home residents will serve as members of the RSG and will be actively involved throughout the study. These PPIE members, who contributed to the development of the original key principles document, will support the recruitment of panel members, assist in interpreting the results from the two rounds of the modified Delphi study and help revise the key principles document.

We recognise the importance of capturing the perspectives of care home residents. Residents, however, have not been directly involved in this study. This is because the modified Delphi survey seeks to develop a set of key principles for care home staff rather than an information resource for care home residents. While the views of residents’ family members may not fully align with those of the resident, we have involved residents’ family members as PPIE contributors as they can provide valuable insights into residents’ needs and experiences.

Indeed, we are mindful that family and friends of care home residents, while not included in the current Delphi process, play an important role in care planning and support.^39^,^41^ To address this, once the modified Delphi survey has been completed, we plan to develop a related resource specifically tailored for residents’ family and friends. Feedback on this resource will be sought through a series of focus groups. Our PPIE members will continue to play a central role in drafting this information resource, recruiting focus group participants and analysing the feedback received.

### Ethics and dissemination

Panel members will receive an email inviting them to take part and a participant information sheet. Panel members must provide their informed consent before completing the first and second rounds of the survey. The study to which this protocol relates was granted ethical approval by the University of Kent’s Division for the Study of Law, Society and Social Justice Research Committee Ethics Panel (reference: 1006) on 9 April 2024.

The results of this project will be disseminated through conferences and one or more peer-reviewed journals presented using the Conducting and REporting of DElphi Studies (CREDES) reporting standard.^16^

## Discussion

This paper details the design of a study using a modified Delphi survey to develop an information resource setting out the key principles to consider when conducting care planning in older adult care homes. The study aims to collect the opinions of people involved in providing care and support within care home settings and gain consensus on a set of key principles which relate to care planning in older adult care homes. The outcomes of this study have the potential to improve care planning for older people living in care homes and could help care home staff to have a better understanding of what person-centred care planning looks like. This is significant as personalised care planning has been found to be more beneficial than usual care for people living with chronic health conditions.^42 43^

There are methodological strengths and weaknesses associated with using a modified Delphi technique. Panel members’ feedback, in the form of written comments and unipolar scale ratings, will help to establish a consensus on the key principles that should inform care planning in older adult care homes. The modified Delphi technique will enable people involved in providing care and support within care home settings from across England to contribute to this study at their convenience. Panellists will remain anonymous, minimising the likelihood that a single individual will dominate the discussion.

One of this study’s potential weaknesses is a large drop-off in the number of participants who complete the second survey. Several methods will be used to maximise the response rate between the first and second surveys. Panellists who complete the survey online can submit their answers in more than one sitting. Panellists will also be given the option to complete the survey over the telephone or by emailing their responses to a member of the research team.

The health and social care services regulator in England, the CQC,^3^ has made it clear that care homes are required to ensure that the people they support are involved in the ‘planning, management and review of their care’. Recent qualitative research, however, has suggested that care planning practices can vary considerably between care home settings.^13^ To improve the consistency of care planning this study design attempts to achieve an expert consensus on the key principles for care planning in older adult care homes. This study aims to produce a set of key principles that will be acceptable to care home practitioners and will promote person-centred care planning. In the future, the RSG intends to share the key principles document developed through this survey with care home residents’ family and friends and invite their feedback through a series of focus groups, with a view to developing a similar resource for residents’ friends and family.

## supplementary material

10.1136/bmjopen-2024-090243 online supplemental file 1

10.1136/bmjopen-2024-090243 online supplemental file 2
