# Supplementary material for: Developing a set of key principles for care planning within older adult care homes: study protocol for a modified Delphi survey
Source: BMJ Open. 2025 Jan 28;15(1):e090243. doi: 10.1136/bmjopen-2024-090243 (PMC11781119; doi:10.1136/bmjopen-2024-090243)
Supplement: online supplemental file 2 [file bmjopen-15-1-s002.pdf]

FUNDED BY
NIHR | National Institute for Health Research

First Block

Care Planning - Key Principles

Thank you so much for your interest in this research project.

Your feedback will be invaluable in helping us to develop a set of key principles which relate to care planning in older adult care homes.

To start with, we need to gather your consent to take part.

Please complete the questions on the next page to proceed.

Participant Consent

Participant Consent Form

I confirm that I have read and understand the information sheet for the study ["Care Planning: Developing a set of key principles", version 3.0, 31 May 2024]. I have had the opportunity to consider the information, ask questions and have had these answered satisfactorily.

- Yes
- No

I understand that my participation is voluntary and that I am free to withdraw at any time, without giving a reason, and without any adverse consequences.

- No
- Yes

I understand who will have access to personal data provided, how the data will be stored and what will happen to the data at the end of the project

- No
- Yes

I understand that information about me would only be disclosed in the very rare circumstance that I or someone else was judged to be at immediate risk of serious harm.

- Yes
- No

I give permission for the researcher(s) to quote me directly [anonymously].

- Yes
- No

I am happy to take part in this research.

- Yes
- No

I consent to be contacted after the second round of the survey has been completed with information about the project. (*optional*)

- Yes
- No

I consent to my data being shared with the UK Data Service (*optional*)

- Yes
- No

Please provide your full name

Please provide your email address
If possible, please do not provide an email address for a shared inbox

Please confirm that you are human.

I'm not a robot
reCAPTCHA
Privacy - Terms

Block 11

Thank you for your interest in this research.

Unfortunately the information you have provided indicates that it would not be appropriate for you to take part. If you would like any further information, please contact Jono Taylor on: jonathan.taylor@ndph.ox.ac.uk

Screening questions

Screening questions

Please complete the following screening questions

I am 18 years of age or older

- Yes
- No

Please indicate how you have been involved in care planning for residents living in an older adult care home in England.

- ☐ Writing care plans
- ☐ Reviewing the contents of care plans
- ☐ Using a care plan as part of providing care and support
- ☐ Supervising care planning
- ☐ Delivering training relating to care planning
- ☐ Contributing to one or more sections of a care plan
- ☐ [ ] Other
- ☐ I am not involved in any way in care planning for residents living in an older adult care home

Please type out "I am answering the screening questions honestly”

[ ]

Opening Page

About this survey

Thank you for your interest in this research project.

When completing this survey please ensure that you can consult the PDF entitled "Key Principles for Care Planning - Round 1"

This survey has seven sections which each relate to a different part of care planning. Please read each statement carefully and answer the questions that follow.

This is the first of two surveys that you will be asked to complete. It is very important that you complete both questionnaires. The reliability of the results could be compromised if people drop out of the study before it is completed, because they feel that the rest of the group does not share their opinions. If people drop out because they feel their opinions are in the minority, the results will overestimate how much the sample of participants agreed on certain aspects of care planning.

This survey should take approximately 25 minutes to complete.

Section 1

This section answers the following question: "What is the purpose of a(n

advanced) care plan?"

Please read through each of the statements carefully before providing feedback.

1. What is the purpose of a care plan?

*An effective care plan provides a **snapshot of a resident's whole life**, including their goals, skills, abilities and how they would like to manage their health and wellbeing.*

How important do you think this statements is?

Not at all important	Slightly important	Moderately important	Very important	Extremely important	I don't know
○	○	○	○	○	○

2. What is the purpose of a care plan?

*When done well, care plans will **empower resident's** to have as much control and independence over their daily life as possible.*

How important do you think this statement is?

Not at all important	Slightly important	Moderately important	Very important	Extremely important	I don't know
○	○	○	○	○	○

3. What is the purpose of a care plan?

The information contained within a strong care plan should help to:

- ***Identify residents' preferences*** *and wishes each time staff provide care or support*

How important do you think this statement is?

Not at all important	Slightly important	Moderately important	Very important	Extremely important	I don't know
○	○	○	○	○	○

4. What is the purpose of a care plan?

The information contained within a strong care plan should help to:

- ***Identify the views of residents*** *or their family and friends regarding the care and support they receive*

How important do you think this statement is?

Not at all important	Slightly important	Moderately important	Very important	Extremely important	I don't know
○	○	○	○	○	○

5. What is the purpose of a care plan?

The information contained within a strong care plan should help to:

- *Maintain* ***continuity of care*** *among external partners and collaborators*

How important do you think this statement is?

Not at all important	Slightly important	Moderately important	Very important	Extremely important	I don't know
○	○	○	○	○	○

6. What is the purpose of a care plan?

The information contained within a strong care plan should help to:

- ***Assess*** *resident's* ***health and wellbeing*** *over time*

How important do you think this statement is?

Not at all important	Slightly important	Moderately important	Very important	Extremely important	I don't know
○	○	○	○	○	○

7. What is the purpose of a care plan?

The information contained within a strong care plan should help to:

- *Assist in **managing staffing levels** and resources*

How important do you think this statement is?

Not at all important	Slightly important	Moderately important	Very important	Extremely important	I don't know
○	○	○	○	○	○

8. What is the purpose of a care plan?

The information contained within a strong care plan should help to:

- *Demonstrate that the **care provided complies with quality-of-care standards***

How important do you think these statements are?

Not at all important	Slightly important	Moderately important	Very important	Extremely important	I don't know
○	○	○	○	○	○

9. What is the purpose of a care plan?

The information contained within a strong care plan should help to:

- *Set out what the **resident's best life** in the home would look like.*

How important do you think these statements are?

Not at all important	Slightly important	Moderately important	Very important	Extremely important	I don't know
○	○	○	○	○	○

10. What is the purpose of an advanced care plan?

*An effective advanced care plan will enable a care home resident to set out their **preferences and priorities for future care**.*

How important do you think this statement is?

Not at all important	Slightly important	Moderately important	Very important	Extremely important	I don't know
○	○	○	○	○	○

11. What is the purpose of an advanced care plan?

Advanced care planning is designed to help ensure that the ***care that people receive in the future is consistent with their values, goals and preferences****.*

How important do you think this statement is?

Not at all important	Slightly important	Moderately important	Very important	Extremely important	I don't know
○	○	○	○	○	○

12. What is the purpose of an advanced care plan?

If not already in place, advanced care planning can lead to the appointment of a ***Lasting Power of Attorney*** *who is legally empowered to make decisions about the treatment a resident would receive if they no longer had the mental capacity to consent.*

How important do you think this statement is?

Not at all important | Slightly important | Moderately important | Very important | Extremely important | I don't know

13. What is the purpose of an advanced care plan?

Advanced care plans often include information about a person's ***end of life care*** *including where the person would like to die, if the person has completed a "do not attempt cardiopulmonary resuscitation" (DNACPR) form, and any, religious and/or spiritual requests.*

How important do you think this statement is?

Not at all important | Slightly important | Moderately important | Very important | Extremely important | I don't know

14. What is the purpose of an advanced care plan?

Advanced care plans may also document a resident's ***future treatment preferences*** *such as whether they want to receive intravenous antibiotics or be admitted to hospital if their condition becomes acute.*

How important do you think this statement is?

Not at all important | Slightly important | Moderately important | Very important | Extremely important | I don't know

Drawing on your knowledge and experience, please use the space below to make any suggestions about this section. You are welcome to comment on:

1. The wording of one or more of the statements
2. The order of the statements
3. Any missing information
4. Any additional comments you may have

Section 2

This section answers the following question: "How can care planning be approached in a person centred way?"

Please read through each of the statements carefully before providing feedback

1. How can care planning be approached in a person centred way?

A ***person-centred care plan*** *will help to ensure that all a resident's needs and preferences are met.*

How important do you think this statement is?

Not at all important	Slightly important	Moderately important	Very important	Extremely important	I don't know
○	○	○	○	○	○

2. How can care planning be approached in a person centred way?

A person-centred care plan has the following qualities:

- It provides a **holistic understanding** of a resident as an individual, including their **history**, **current interests** and **future ambitions**. It will detail:
  - *The health, social and emotional issues for which a resident requires support*

How important do you think these statements are?

Not at all important	Slightly important	Moderately important	Very important	Extremely important	I don't know
○	○	○	○	○	○

3. How can care planning be approached in a person centred way?

A person-centred care plan has the following qualities:

- It provides a **holistic understanding** of a resident as an individual, including their **history**, **current interests** and **future ambitions**. It will detail:
  - *The resident's personal values and priorities for their care*

How important do you think these statements are?

Not at all important	Slightly important	Moderately important	Very important	Extremely important	I don't know
○	○	○	○	○	○

4. How can care planning be approached in a person centred way?

A person-centred care plan has the following qualities:

- It provides a **holistic understanding** of a resident as an individual, including their **history**, **current interests** and **future ambitions**. It will detail:
  - *The resident's capabilities as well their needs*

How important do you think these statements are?

Not at all important	Slightly important	Moderately important	Very important	Extremely important	I don't know
○	○	○	○	○	○

5. How can care planning be approached in a person centred way?

A person-centred care plan has the following qualities:

- It **engages the resident**, and **key stakeholders**, in **decision-making**. This can be achieved by:
  - *Inviting residents to take the lead in discussing the care plan's contents, wherever possible*

How important do you think these statements are?

Not at all important	Slightly important	Moderately important	Very important	Extremely important	I don't know
○	○	○	○	○	○

6. How can care planning be approached in a person centred way?

A person-centred care plan has the following qualities:

- It **engages the resident**, and **key stakeholders**, in **decision-making**. This can be achieved by:
  - *Taking reasonable steps to meet residents' communication (e.g., plain English, information available in Braille, translators) and sensory needs (e.g., hearing aids, glasses).*

How important do you think these statements are?

Not at all important	Slightly important	Moderately important	Very important	Extremely important	I don't know
○	○	○	○	○	○

7. How can care planning be approached in a person centred way?

A person-centred care plan has the following qualities:

- It **engages the resident**, and **key stakeholders**, in **decision-making**. This can be achieved by:
  - *Including input from important people in the resident's life*

How important do you think these statements are?

Not at all important	Slightly important	Moderately important	Very important	Extremely important	I don't know
○	○	○	○	○	○

8. How can care planning be approached in a person centred way?

A person-centred care plan has the following qualities:

- It **engages the resident**, and **key stakeholders**, in **decision-making**. This can be achieved by:
  - *Ensuring that residents and their family and friends are aware of all the available options and providing them with the information necessary to make informed decisions*

How important do you think these statements are?

Not at all important	Slightly important	Moderately important	Very important	Extremely important	I don't know
○	○	○	○	○	○

9. How can care planning be approached in a person centred way?

A person-centred care plan has the following qualities:

- It **engages the resident**, and **key stakeholders**, in **decision-making**. This can be achieved by:
  - *Including input from external care providers, professionals and organisations involved in promoting the resident's health and wellbeing*

How important do you think these statements are?

Not at all important	Slightly important	Moderately important	Very important	Extremely important	I don't know
○	○	○	○	○	○

Drawing on your knowledge and experience, please use the space below to make any suggestions about this section. You are welcome to comment on:

1. The wording of one or more of the statements,
2. The order of the statements
3. Any missing information
4. Any additional comments you may have

Section 3

This section answers the following question: "What should be contained within a care plan?"

Please read through each of the statements carefully before providing feedback.

1. What should be contained within a care plan?

Care plans will contain different sections. High quality care plans are likely to include:

- *A recent* ***photograph*** *of the resident*

How important do you think these statements are?

Not at all important	Slightly important	Moderately important	Very important	Extremely important	I don't know
○	○	○	○	○	○

2. What should be contained within a care plan?

Care plans will contain different sections. High quality care plans are likely to include:

- **Details** about the **care plan itself**:
  - *A record of when the plan has been created, reviewed, updated and modified and when the care plan will next be reviewed.*

How important do you think these statements are?

Not at all important	Slightly important	Moderately important	Very important	Extremely important	I don't know
○	○	○	○	○	○

4. What should be contained within a care plan?

Care plans will contain different sections. High quality care plans are likely to include:

- Background information about the **resident's history**, including details of:
    - *The resident's life immediately prior to moving into the care home*

How important do you think these statements are?

Not at all important	Slightly important	Moderately important	Very important	Extremely important	I don't know
○	○	○	○	○	○

5. What should be contained within a care plan?

Care plans will contain different sections. High quality care plans are likely to include:

- Background information about the **resident's history**, including details of:
    - *The resident's family, culture and religion*

How important do you think these statements are?

Not at all important	Slightly important	Moderately important	Very important	Extremely important	I don't know
○	○	○	○	○	○

6. What should be contained within a care plan?

Care plans will contain different sections. High quality care plans are likely to include:

- Background information about the **resident's history**, including details of:
  - *Key dates and life events, such as significant holidays, birthdays or anniversaries*

How important do you think these statements are?

Not at all important	Slightly important	Moderately important	Very important	Extremely important	I don't know
○	○	○	○	○	○

7. What should be contained within a care plan?

Care plans will contain different sections. High quality care plans are likely to include:

- Information about a resident's **hobbies, interests and aspirations, past and present**:

- *Information about how to support the resident's current goals*

How important do you think these statements are?

Not at all important	Slightly important	Moderately important	Very important	Extremely important	I don't know
○	○	○	○	○	○

8. What should be contained within a care plan?

Care plans will contain different sections. High quality care plans are likely to include:

- Information about a resident's **hobbies, interests and aspirations, past and present**:
    - *Information about activities the resident would/would not like to take part in*

How important do you think these statements are?

Not at all important	Slightly important	Moderately important	Very important	Extremely important	I don't know
○	○	○	○	○	○

9. What should be contained within a care plan?

Care plans will contain different sections. High quality care plans are likely to include:

- Information about the **risks that the resident may face**, and steps that can be taken to mitigate them in a person centred way

How important do you think these statements are?

Not at all important	Slightly important	Moderately important	Very important	Extremely important	I don't know
○	○	○	○	○	○

10. What should be contained within a care plan?

Care plans will contain different sections. High quality care plans are likely to include:

- Information about **forthcoming appointments** and details of who will be responsible for arranging transportation and accompanying the resident, these could be medical or social appointments

How important do you think these statements are?

Not at all important	Slightly important	Moderately important	Very important	Extremely important	I don't know
○	○	○	○	○	○

11. What should be contained within a care plan?

Care plans will contain different sections. High quality care plans are likely to include:

- Information about the **resident's health**, including, but not limited to:
  - ***Vital signs***

How important do you think these statements are?

Not at all important	Slightly important	Moderately important	Very important	Extremely important	I don't know
○	○	○	○	○	○

12. What should be contained within a care plan?

Care plans will contain different sections. High quality care plans are likely to include:

- Information about the **resident's health**, including, but not limited to:
  - ***Medication***

How important do you think these statements are?

Not at all important	Slightly important	Moderately important	Very important	Extremely important	I don't know
○	○	○	○	○	○

13. What should be contained within a care plan?

Care plans will contain different sections. High quality care plans are likely to include:

- Information about the **resident's health**, including, but not limited to:
  - ***Dietary*** *and* ***hydration needs***

How important do you think these statements are?

Not at all important	Slightly important	Moderately important	Very important	Extremely important	I don't know
○	○	○	○	○	○

14. What should be contained within a care plan?

Care plans will contain different sections. High quality care plans are likely to include:

- Information about the **resident's health**, including, but not limited to:
  - *History of **physical, mental and oral health***

How important do you think these statements are?

Not at all important	Slightly important	Moderately important	Very important	Extremely important	I don't know
○	○	○	○	○	○

15. What should be contained within a care plan?

Care plans will contain different sections. High quality care plans are likely to include:

- Information about the **resident's day-to-day care needs and preferences**, including:
  - *The **resident's capability** to meet, and their preferences for receiving support for, their day-to-day needs*

How important do you think these statements are?

Not at all important	Slightly important	Moderately important	Very important	Extremely important	I don't know
○	○	○	○	○	○

16. What should be contained within a care plan?

Care plans will contain different sections. High quality care plans are likely to include:

- Information about the **resident's day-to-day care needs and preferences**, including:
    - *Details of any **specialist equipment** that the resident may need, such as adapted cutlery or mobility aids*

How important do you think these statements are?

Not at all important	Slightly important	Moderately important	Very important	Extremely important	I don't know
○	○	○	○	○	○

17. What should be contained within a care plan?

Care plans will contain different sections. High quality care plans are likely to include:

- Information about a resident's **end of life care**, including:
    - *Where the resident would like to be cared for*

How important do you think these statements are?

Not at all important	Slightly important	Moderately important	Very important	Extremely important	I don't know
○	○	○	○	○	○

18. What should be contained within a care plan?

Care plans will contain different sections. High quality care plans are likely to include:

- Information about a resident's **end of life care**, including:
  - *Details of religious, spiritual and/or cultural practices*

How important do you think these statements are?

Not at all important	Slightly important	Moderately important	Very important	Extremely important	I don't know
○	○	○	○	○	○

19. What should be contained within a care plan?

Care plans will contain different sections. High quality care plans are likely to include:

- Information about a resident's **end of life care**, including:
  - *Key people to involve*

How important do you think these statements are?

Not at all important	Slightly important	Moderately important	Very important	Extremely important	I don't know
○	○	○	○	○	○

20. What should be contained within a care plan?

Care plans will contain different sections. High quality care plans are likely to include:

- Information about a resident's **end of life care**, including:
  - *Who the resident would like to be with them in their final moments*

How important do you think these statements are?

Not at all important	Slightly important	Moderately important	Very important	Extremely important	I don't know
○	○	○	○	○	○

21. What should be contained within a care plan?

Care plans will contain different sections. High quality care plans are likely to include:

- Information about a resident's **end of life care**, including:
    - *Palliative medical care and resuscitation preferences*

How important do you think these statements are?

Not at all important	Slightly important	Moderately important	Very important	Extremely important	I don't know
○	○	○	○	○	○

22. What should be contained within a care plan?

Care plans will contain different sections. High quality care plans are likely to include:

- Information about a resident's **end of life care**, including:
    - *Funeral arrangements*

How important do you think these statements are?

Not at all important	Slightly important	Moderately important	Very important	Extremely important	I don't know
○	○	○	○	○	○

Drawing on your knowledge and experience, please use the space below to make any suggestions about this section. You are welcome to comment on:

1. The wording of one or more of the statements,
2. The order of the statements
3. Any missing information
4. Any additional comments you may have

Section 4

This section answers the following question: "When will a care plan be developed and updated?"

Please read through each of the statements carefully before providing feedback.

1. When will a care plan be developed and updated?

***Prior to**, or **shortly after**, a **person begins residence** at a care home.*

How important do you think this statement is?

Not at all important	Slightly important	Moderately important	Very important	Extremely important	I don't know
○	○	○	○	○	○

2. When will a care plan be developed and updated?

*Where possible, **key information** about a resident (such as their health conditions and medical needs) should be **included in a care plan prior to their admission** to a care home.*

How important do you think this statement is?

Not at all important	Slightly important	Moderately important	Very important	Extremely important	I don't know
○	○	○	○	○	○

3. When will a care plan be developed and updated?

*Information collected prior to someone being admitted to a care home - which could be collected as part of a pre-admission assessment - may be obtained by **talking to** the **resident** and/or their **family**, **friends**, their **General Practitioner** (GP) or **social worker**.*

How important do you think this statement?

Not at all important	Slightly important	Moderately important	Very important	Extremely important	I don't know
○	○	○	○	○	○

4. When will a care plan be developed and updated?

*In the **first 2-4 weeks** following a person's arrival at a care home, as staff begin to get to know the resident better, it is often helpful to **set aside time to develop a care plan**.*

How important do you think this statement is?

Not at all important	Slightly important	Moderately important	Very important	Extremely important	I don't know
○	○	○	○	○	○

5. When will a care plan be developed and updated?

Thereafter, an effective ***care plan will be routinely updated****, possibly in the form of regular and extensive reviews, to ensure the document reflects a resident's current needs and interests.*

How important do you think this statement is?

Not at all important	Slightly important	Moderately important	Very important	Extremely important	I don't know
○	○	○	○	○	○

6. When will a care plan be developed and updated?

To ensure that care plans remain accurate and up-to-date, ***regular reviews*** *are likely to take place* ***at least every six weeks****.*

How important do you think this statement is?

Not at all important	Slightly important	Moderately important	Very important	Extremely important	I don't know
○	○	○	○	○	○

7. When will a care plan be developed and updated?

Regular reviews *can provide an opportunity to* ***assess***

*the **contents** of a resident's care plan and **discuss** whether any **changes** need to be made.*

How important do you think this statement is?

Not at all important	Slightly important	Moderately important	Very important	Extremely important	I don't know
○	○	○	○	○	○

8. When will a care plan be developed and updated?

*More **detailed** care plan **reviews** may take place **every six months**, and where possible, **include family members***

How important do you think this statement is?

Not at all important	Slightly important	Moderately important	Very important	Extremely important	I don't know
○	○	○	○	○	○

9. When will a care plan be developed and updated?

*A care plan should also be updated in response to **significant changes** or **incidents** in a resident's life such as a **fall**, a **deterioration** in a their mental and physical **health**, or a **hospital admission**.*

How important do you think this statement is?

Not at all important	Slightly important	Moderately important	Very important	Extremely important	I don't know
○	○	○	○	○	○

Above, in statement 6, we have suggested that **regular reviews of care plans** are likely to take place at least every **six weeks**.

How often do you think these regular reviews should take place?

- ○ Once a week
- ○ Once every two weeks
- ○ Once every three weeks
- ○ Once every four weeks
- ○ Once every five weeks
- ○ Once every six weeks
- ○ Once every seven weeks
- ○ Once every eight weeks
- ○ Other
- ○ I don't know

Above, in statement 8, we have suggested that more **detailed care plan reviews** may take place every **six months**.

How often do you think these more detailed care plan reviews should take place?

- ◯ Once a month
- ◯ Once every two months
- ◯ Once every three months
- ◯ Once every four months
- ◯ Once every five months
- ◯ Once every six months
- ◯ Once every seven months
- ◯ Once every eight months
- ◯ Other [ ]
- ◯ I don't know

Drawing on your knowledge and experience, please use the space below to make any suggestions about this section. You are welcome to comment on:

1. The wording of one or more of the statements,
2. The order of the statements
3. Any missing information
4. Any additional comments you may have

Section 5

This section answers the following question: "Who is likely to contribute to a care plan?"

Please read through each of the statements carefully before providing feedback.

1. Who is likely to contribute to a care plan?

- *Where possible, **residents should be involved** in developing and reviewing their care plans*

How important do you think this statement is?

Not at all important	Slightly important	Moderately important	Very important	Extremely important	I don't know
○	○	○	○	○	○

2. Who is likely to contribute to a care plan?

- ***People** who are **important to residents**, including their **family** and **friends**, should be involved*

How important do you think this statement is?

Not at all important	Slightly important	Moderately important	Very important	Extremely important	I don't know
○	○	○	○	○	○

3. Who is likely to contribute to a care plan?

- ***Senior care*** *or* ***nursing staff*** *are usually responsible for writing care plans; however, valuable information can also be provided by* ***front line care workers*** *and* ***non-care staff*** *- such as members of the* ***housekeeping*** *and* ***catering teams****.*

How important do you think this statement is?

Not at all important	Slightly important	Moderately important	Very important	Extremely important	I don't know
○	○	○	○	○	○

4. Who is likely to contribute to a care plan?

- ***External health*** *and* ***care professionals****, such as* ***medical consultants****,* ***social workers****,* ***GPs****, and* ***occupational therapists****, may contribute to specific parts of the care plan.*

How important do you think this statement is?

Not at all important	Slightly important	Moderately important	Very important	Extremely important	I don't know
○	○	○	○	○	○

Drawing on your knowledge and experience, please use the space below to make any suggestions about this section. You are welcome to comment on:

1. The wording of one or more of the statements,
2. The order of the statements
3. Any missing information
4. Any additional comments you may have

Section 6

This section answers the following question: "Who should have access to a care plan?"

Please read through each of the statements carefully before providing feedback.

1. Who should have access to a care plan?

To be most useful, care plans will need to be **accessible to**:

- *The residents themselves*

How important do you think this statement is?

Not at all important	Slightly important	Moderately important	Very important	Extremely important	I don't know
○	○	○	○	○	○

2. Who should have access to a care plan?

To be most useful, care plans will need to be **accessible to**:

- *People who have legal power of attorney for the care home resident*

How important do you think this statement is?

Not at all important	Slightly important	Moderately important	Very important	Extremely important	I don't know
○	○	○	○	○	○

3. Who should have access to a care plan?

To be most useful, care plans will need to be **accessible to**:

- *Members of a resident's 'circle of care', such as family and friends nominated by the resident*

How important do you think this statement is?

Not at all important	Slightly important	Moderately important	Very important	Extremely important	I don't know
○	○	○	○	○	○

4. Who should have access to a care plan?

To be most useful, care plans will need to be **accessible to**:

- *Care home staff, including bank and agency staff*

How important do you think this statement is?

Not at all important	Slightly important	Moderately important	Very important	Extremely important	I don't know
○	○	○	○	○	○

5. Who should have access to a care plan?

To be most useful, care plans will need to be **accessible to**:

- *External health and care professionals, such as social workers, GPs, and pharmacists*

How important do you think this statement is?

Not at all important	Slightly important	Moderately important	Very important	Extremely important	I don't know
○	○	○	○	○	○

Drawing on your knowledge and experience, please use the space below to make any suggestions about this section. You are welcome to comment on:

1. The wording of one or more of the statements,
2. The order of the statements
3. Any missing information

4. Any additional comments you may have

Section 7

This section relates to the following topic: "Future developments in care planning"

Please read through each of the statements carefully before providing feedback.

1. Future developments in care planning

Technology, such as digital care planning software, is **playing an increasingly important role** in supporting care planning. **Digital care plans** can:

- *Help to reduce the amount of time to complete care plans*

How important do you think this statement is?

Not at all important	Slightly important	Moderately important	Very important	Extremely important	I don't know
○	○	○	○	○	○

2. Future developments in care planning

Technology, such as digital care planning software, is **playing an increasingly important role** in supporting care planning. **Digital care plans** can:

- *Improve staff engagement in care planning*

How important do you think this statement is?

Not at all important	Slightly important	Moderately important	Very important	Extremely important	I don't know
○	○	○	○	○	○

3. Future developments in care planning

Technology, such as digital care planning software, is **playing an increasingly important role** in supporting care planning. **Digital care plans** can:

- *Produce aggregate data which can help the home plan for the future*

How important do you think this statement is?

Not at all important	Slightly important	Moderately important	Very important	Extremely important	I don't know
○	○	○	○	○	○

4. Future developments in care planning

Technology, such as digital care planning software, is **playing an increasingly important role** in supporting care planning. **Digital care plans** can:

- *Allow information to be securely and quickly shared with relevant stakeholders, such as health and social care professionals and a person's family and/or friends*

How important do you think this statement is?

Not at all important	Slightly important	Moderately important	Very important	Extremely important	I don't know
○	○	○	○	○	○

5. Future developments in care planning

Care homes that are interested in adopting digital care plans may **need to consider**:

- *Whether they have the necessary resources to purchase the software licences and accompanying electronic devices*

How important do you think this statement is?

Not at all important	Slightly important	Moderately important	Very important	Extremely important	I don't know
○	○	○	○	○	○

6. Future developments in care planning

Care homes that are interested in adopting digital care plans may **need to consider**:

- *If the software selected allows staff to develop person-centred care plans*

How important do you think this statement is?

Not at all important	Slightly important	Moderately important	Very important	Extremely important	I don't know
○	○	○	○	○	○

7. Future developments in care planning

Care homes that are interested in adopting digital care plans may **need to consider**:

- *Whether they have sufficient internet coverage across their site(s)*

How important do you think this statement is?

Not at all important	Slightly important	Moderately important	Very important	Extremely important	I don't know
○	○	○	○	○	○

8. Future developments in care planning

Care homes that are interested in adopting digital care plans may **need to consider**:

- *Whether the digital care plan can be made accessible to all the relevant people involved in supporting the resident, while ensuring that only appropriate people will be able to update the digital care plan*

How important do you think this statement is?

Not at all important	Slightly important	Moderately important	Very important	Extremely important	I don't know
○	○	○	○	○	○

9. Future developments in care planning

Care homes that are interested in adopting digital care plans may **need to consider**:

- *The time commitment likely to be associated with transitioning from paper to digital care plans*

How important do you think this statement is?

Not at all important	Slightly important	Moderately important	Very important	Extremely important	I don't know
○	○	○	○	○	○

10. Future developments in care planning

Care homes that are interested in adopting digital care plans may **need to consider**:

- *The time commitment likely to be associated with training and supporting staff to use digital care*

planning packages as well as meeting ongoing training needs

How important do you think this statement is?

Not at all important	Slightly important	Moderately important	Very important	Extremely important	I don't know
○	○	○	○	○	○

Drawing on your knowledge and experience, please use the space below to make any suggestions about this section. You are welcome to comment on:

1. The wording of one or more of the statements,
2. The order of the statements
3. Any missing information
4. Any additional comments you may have

Additional comments

Additional comments

Please add any final comments you wish to make in the box below.

Respondents' details

Your details

Finally, please could you provide us with some information about yourself.

How do you identify yourself?

- Male
- Female
- Non-binary/third gender
- Prefer to self-describe as
- Prefer not to say

Please indicate your age by selecting one of the categories below:

- ○ 18 - 24
- ○ 25 - 34
- ○ 35 - 44
- ○ 45 - 54
- ○ 55 - 64
- ○ 65 - 74
- ○ 75 or older
- ○ Prefer not to say

What is your ethnic group?

- ○ White: English/Welsh/Scottish/Northern Irish/British
- ○ White: Irish
- ○ White: Gypsy or Irish Traveller
- ○ White: Any other White background, please describe
- ○ Mixed/Multiple: White and Black Caribbean
- ○ Mixed/Multiple: White and Black African
- ○ Mixed/Multiple: White and Asian
- ○ Mixed/Multiple: Any other Mixed/Multiple ethnic background, please describe
- ○ Asian/Asian British: Indian
- ○ Asian/Asian British: Pakistani

- ○ Asian/Asian British: Bangladeshi
- ○ Asian/Asian British: Chinese
- ○ [ ] Any other Asian background, please describe
- ○ Black/ African/Caribbean/Black British
- ○ Black/ African/Caribbean/Black British: Caribbean
- ○ Black/ African/Caribbean/Black British: Any other Black/African/Caribbean background, please describe [ ]
- ○ Other ethnic group: Arab
- ○ Other ethnic group: Any other ethnic group, please describe [ ]
- ○ Prefer not to say

How long have you been involved in supporting the older adult care home sector?

- ○ Up to 1 year
- ○ 1 - 2 years
- ○ 2 - 5 years
- ○ 5 - 10 years
- ○ More than 10 years
- ○ Prefer not to say

In what organisation(s) have/are you involved in care

planning?

- ☐ Care home
- ☐ Nursing home
- ☐ Dual-registered care home
- ☐ Third sector organisation (e.g. National Care Forum)
- ☐ Regulator of health and social care (e.g. Care Quality Commission)
- ☐ Professional body (e.g. National Care Association)
- ☐ Prefer not to say
- ☐ [ ] Other

When you have been involved in care planning what was/is your job title?

- ○ [ ] Job title(s):
- ○ Prefer not to say

Powered by Qualtrics
