# Supplementary material for: Developing a set of key principles for care planning within older adult care homes: study protocol for a modified Delphi survey
Source: BMJ Open. 2025 Jan 28;15(1):e090243. doi: 10.1136/bmjopen-2024-090243 (PMC11781119; doi:10.1136/bmjopen-2024-090243)
Supplement: Supplementary file 3 [file bmjopen-2024-090243.reviewer_comments.pdf]

PEER REVIEW HISTORY

BMJ Open publishes all reviews undertaken for accepted manuscripts. Reviewers are asked to complete a checklist review form and are provided with free text boxes to elaborate on their assessment. These free text comments are reproduced below.

ARTICLE DETAILS

Title (Provisional)

Developing a set of key principles for care planning within older adult care homes: study protocol for a modified Delphi survey

Authors

Taylor, Jonathan; Caprioli, Thais; Damant, Jacqueline; Hamashima, Yuri; Jasim, Sarah; Smith, Nick; Toma, Madalina

---

VERSION 1 - REVIEW

**Reviewer**	**1**
**Name**	**Kimpel, Christine**
**Affiliation**	**Vanderbilt University, Nursing**
**Date**	**30-Aug-2024**
**COI**	**None declared.**

---

1. Thank you for the opportunity to review this protocol on an important and timely topic. Overall, I found this report well-written, clear, and informative. I have offered some comments for the authors' consideration.

2. Abstract: The abstract provided clear and detailed information on the study protocol.

3. Introduction and research objective: The study objective was appropriate as a next step to build on the scoping review, which revealed variations in care planning procedures across care homes.

4. Under “justification for study design,” the authors may want to consider changing the term “stakeholders” to “partners, collaborators, etc.” The term has a background in colonialism and the marginalization of indigenous populations. Additionally, it lacks specificity, and the protocol may benefit from examples of care home staff roles: leaders, caregivers, nurses, etc.

5. Under “justification for study design,” I appreciated the explanation of how the modified Delphi approach would address bias.

6. “Research steering committee”: The authors may consider specifying a range for the expected number of committee members.

7. “Research steering committee”: Since the RSG will draft the principles, the authors may want to include how they draft the key principles document and steps that will be taken to avoid introducing bias in this phase of the process.

8. “Eligibility Criteria”: To ensure adequate expertise, the authors may benefit from including a stipulation that participants have a minimum of one year of experience in a role that involves care planning.

9. “Survey development”: To keep this section consistent with the abstract, I recommend including the numeral rating for the Likert scale, e.g., “not at all important” = 1.

10. “Definition of consensus”: Same comment as the previous item.

11. “First round”: The authors have clearly explained how to minimize missing data and inaccurate responses. To convey the participant's cognitive burden, I suggest adding an expected number of survey items/questions.

12. “Second round”: Will providing panel members with a copy of their round 1 responses possibly bias their round 2 responses? The authors may want to consider explaining this decision.

**Reviewer**	**2**
**Name**	**Vander Stichele, Robert**
**Affiliation**	**Universiteit Gent, Unit of clinical Pharmacology**
**Date**	**08-Oct-2024**
**COI**	**none**

This is a protocol for a Delphi study on what should be the key principles for a care plan in care homes.

It is not clear how the statements were generated and whether they cover the domain.

The difference between care plan and advance care plan is not clearly set out.

Likert scales are bipolar scalings of opinions of respondents' opinions (agree or disagree). Here an unipolar scale of importance is presented (from 0 to 4). There is a difference between asking whether an item on a list is key and asking respondents to express their opinion about a statement.

The patient involvement is not sufficient.

The recruitment procedure does not guarantee a multidisciplinary composition of the panel.

VERSION 1 - AUTHOR RESPONSE

Reviewer 1, Dr. Christine Kimpel, Vanderbilt University

1. **Thank you for the opportunity to review this protocol on an important and timely topic. Overall, I**

found this report well-written, clear, and informative. I have offered some comments for the authors' consideration.

Many thanks for your positive feedback.

2. Abstract: The abstract provided clear and detailed information on the study protocol.

Many thanks for your positive feedback.

3. Introduction and research objective: The study objective was appropriate as a next step to build on the scoping review, which revealed variations in care planning procedures across care homes.

Many thanks for your positive feedback.

4. Under "justification for study design," the authors may want to consider changing the term "stakeholders" to "partners, collaborators, etc." The term has a background in colonialism and the marginalization of indigenous populations. Additionally, it lacks specificity, and the protocol may benefit from examples of care home staff roles: leaders, caregivers, nurses, etc.

Thank you for raising this issue, we have now removed the word stakeholder from the manuscript and have replaced it with the following phrase "people involved in providing care and support within care home settings".

The "justification for study design" section now also includes some more information about the job titles of the people involved in the earlier consultation work.

5. Under "justification for study design," I appreciated the explanation of how the modified Delphi approach would address bias.

Thank you for raising this issue, the "justification for study design" now includes an explanation of how the modified Delphi will seek to minimize potential bias.

6. "Research steering committee": The authors may consider specifying a range for the expected number of committee members.

Thank you for raising this issue, we have now mentioned the number of people on the RSC.

7. “Research steering committee”: Since the RSG will draft the principles, the authors may want to include how they draft the key principles document and steps that will be taken to avoid introducing bias in this phase of the process.

Thank you for raising this issue, the manuscript has now been revised to explain the steps that will be taken to avoid introducing bias in this phase of the process. These changes can be found in the section entitled “Survey development”.

8. “Eligibility Criteria”: To ensure adequate expertise, the authors may benefit from including a stipulation that participants have a minimum of one year of experience in a role that involves care planning.

Thank you very much for raising this point. This is something that the RSC discussed at some length prior to submitting our manuscript. In the end, we decided not to include this requirement because we felt it would be helpful to receive feedback from participants at different stages in their carer, including those who had worked in the care home sector for fewer than 12 months. As part of the survey, participants will be asked how many months/years that have been providing care and support to care home residents and we plan to consider this data when analyzing participants’ responses.

9. “Survey development”: To keep this section consistent with the abstract, I recommend including the numeral rating for the Likert scale, e.g., “not at all important” = 1.

Thank you for raising this issue. The manuscript has been revised accordingly.

10. “Definition of consensus”: Same comment as the previous item.

Thank you for raising this issue. The manuscript has been revised accordingly.

11. “First round”: The authors have clearly explained how to minimize missing data and inaccurate responses. To convey the participant's cognitive burden, I suggest adding an expected number of survey items/questions.

Thank you for raising this issue. The manuscript has been revised accordingly.

12. “Second round”: Will providing panel members with a copy of their round 1 responses possibly bias their round 2 responses? The authors may want to consider explaining this decision.

Thank you for raising this issue, we decided to provide panel members with a copy of their round 1 responses as this approach is consistent with previous Delphi studies. As well as their own responses, panel members will be provided with an anonymized summary of the other participants’ responses. The anonymity of this information is designed to help reduce bias as participants will not be aware of the social or professional status of the other panel members.

In addition to this information, where the wording of statements has been changed, participants will be provided with an explanation of why the changes have been made. These explanations will draw on participants' feedback gathered during the first round of the Delphi study.

The manuscript has been revised to make this information clearer to the reader.

Reviewer 2: Dr. Robert Vander Stichele, Universiteit Gent

This is a protocol for a Delphi study on what should be the key principles for a care plan in care homes.

It is not clear how the statements were generated and whether they cover the domain.

Thank you for raising this issue. The RSC began by drafting a single document which set out a series of key principles relating to care planning. The contents of this document were informed by:

1. Consultations with people involved in providing care and support within care home settings
2. A systematic scoping review
3. Ongoing input from the relatives of two care home residents who were previously involved in activities (1) and (2)

Once drafted, the contents of this document were turned into a series of statements which comprised a single sentence or bullet point. The key principles and the survey will then be (4) piloted with two care home managers who meet the eligibility criteria. Once these individuals had read through the draft document and completed the survey, they will met with two members of the RSC to share their feedback. The document and the survey will be revised in light of feedback obtained during the pilot. These care home managers will not take part in the final modified Delphi surveys and their scores on the draft survey will not be included in our analysis of round 1 and 2 of the modified Delphi surveys. Copies of the pilot document and the pilot survey have been provided as supplementary files 1 and 2.

As part of the Delphi survey, panelists will be presented with the full document as a PDF attachment and will then be presented with the constituent statements in the online survey.

The manuscript has now been revised to make these steps clearer in the section entitled "Survey development".

The difference between care plan and advance care plan is not clearly set out.

Thank you for raising this issue, we have now updated the manuscript to include a fuller explanation of the difference between care planning and advance care planning on page two of the manuscript.

Likert scales are bipolar scalings of opinions of respondents' opinions (agree or disagree). Here an unipolar scale of importance is presented (from 0 to 4). There is a difference between asking whether an item on a list is key and asking respondents to express their opinion about a statement.

Thank you for raising this issue. The RSC spent a great deal of time discussing:

- What questions to include in the survey
- The number of points (particularly whether to include 5 or 7) to include on the scale
- Whether to include a "I don't know" option.

In the end, we decided to present survey participants with a 5-point scale as well as a "I don't know" option. The exact wording of this scale was informed by previous Delphi studies.

For the first Delphi survey, participants will also be invited to comment on each of the statements. Participants will be told that their comments can relate to: The wording of the statement, the order of the statements, any missing information, any additional comments they may have.

The manuscript has been revised to make this information clearer to the reader. These changes can be
found in the section entitled "Survey development".

The patient involvement is not sufficient.

Thank you for raising this issue. The RSC considers patient and public involvement and engagement involvement to be a crucial part of developing the key principles. This project has involved two dedicated PPIE members whose family members have spent time in a number of care homes. These PPIE members assisted in the previous work schemes (the consultations with care home managers and the systematic scoping review) whose findings have informed the current project. These individuals will be involved in all aspects of the Delphi study – from helping to draft the original key principles and developing the recruitment strategy, to analyzing the feedback and revising the key principles accordingly.

While this Delphi study is concerned with developing a resource of care home staff, we recognize the value that residents' friends and family members can play. A future scheme of work will develop a resource for this group and gather their feedback through a series of focus groups. Our PPIE members will play a key role in this work.

The manuscript has been revised to clarify the role that PPIE members will play in this project and plans for a related scheme of work which will directly engage with care home residents' friends and family members.

The recruitment procedure does not guarantee a multidisciplinary composition of the panel.

Thank you for highlighting this point. The recruitment section of the paper has been updated to outline additional organizations that will be approached to enhance the likelihood of establishing a multidisciplinary panel.

VERSION 2 - REVIEW

**Reviewer**	**1**
**Name**	**Kimpel, Christine**
**Affiliation**	**Vanderbilt University, Nursing**
**Date**	**26-Nov-2024**
**COI**	

---

1. Thank you for the opportunity to review this manuscript for a second time. I found the clarity of reporting to be much improved in this revision.

2. In the “Survey Development” section, I appreciate that the authors addressed how to limit bias in this stage by pre-testing the survey with two people involved in care-planning. They stated that these two people must meet “eligibility criteria.” Please clarify which criteria they must meet. Thank you!

3. “First round,”since respondents will have the option to state “I don’t know,” they authors may want explain what they will do in the event the “I don’t know” is used for a majority of the items and/or across a majority of the respondents.

4. Anywhere that “Likert” scale is used: Per the second reviewer from the first round, please consider changing Likert (which is a bipolar scale), to “unipolar” which reflects the proposed response items.

5. I agree with the second reviewer from the previous review that the patient representation in this process is not sufficient. While the authors have been mindful of including family members of residents in the process, their perspectives differ from those of residents in care homes. I found a systematic review regarding the inclusion of patients within the Delphi process and the benefits. I highly recommend the authors reconsider their protocol to enhance patient involvement: https://bmjopen.bmj.com/content/11/9/e051066 (“Patient participation in Delphi surveys to develop core outcome sets: systematic review”). If not, the authors may consider highlighting this as a major limitation of the study and providing a clear rationale for why patients are not involved in this process. Thank you again.

VERSION 2 - AUTHOR RESPONSE

<u>Reviewer 1: Dr. Christine Kimpel, Vanderbilt University</u>

1. Thank you for the opportunity to review this manuscript for a second time. I found the clarity of reporting to be much improved in this revision.

Many thanks for your positive feedback.

2. In the “Survey Development” section, I appreciate that the authors addressed how to limit bias in this stage by pre-testing the survey with two people involved in care-planning. They stated that these two people must meet “eligibility criteria.” Please clarify which criteria they must meet. Thank you!

We are sorry for the misunderstanding; the two people who will be involved in piloting the survey will be required to meet the same eligibility criteria as the round one panel members, details of which can be found in the “Eligibility criteria” subsection. The manuscript has been revised to make this clearer to the reader.

3. “First round,” since respondents will have the option to state “I don’t know,” they authors may want explain what they will do in the event the “I don’t know” is used for a majority of the items and/or across a majority of the respondents.

Thank you for raising this issue. The “Analysis of round one responses” and the “Analysis of round two responses” subsections have been revised to include details of steps that will be

taken if “I don’t know” is used for a majority of the items and/or across a majority of the respondents. These revisions can be found on page 8 of the manuscript.

4. Anywhere that “Likert” scale is used: Per the second reviewer from the first round, please consider changing Likert (which is a bipolar scale), to “unipolar” which reflects the proposed response items.

Thank you for raising this issue. We have now replaced the term “Likert scale” with “unipolar scale” throughout the manuscript.

5. I agree with the second reviewer from the previous review that the patient representation in this process is not sufficient. While the authors have been mindful of including family members of residents in the process, their perspectives differ from those of residents in care homes. I found a systematic review regarding the inclusion of patients within the Delphi process and the benefits. I highly recommend the authors reconsider their protocol to enhance patient involvement: https://bmjopen.bmj.com/content/11/9/e051066 (“Patient participation in Delphi surveys to develop core outcome sets: systematic review”). If not, the authors may consider highlighting this as a major limitation of the study and providing a clear rationale for why patients are not involved in this process. I will defer to the editors final decision regarding this feedback. Thank you again.

Thank you for raising this issue and for drawing our attention to the article by Barrington et al. We agree that direct inclusion of care home residents in the Delphi process can offer unique insights; however, after careful consideration, we decided not to include care home residents in this study because the primary aim of our document is to support care home staff, making them the key focus of this work. While care home residents are certainly affected by the topics discussed, the document is not directly intended for their use.

While we have included family members of residents in the Delphi process to ensure the residents’ perspectives are indirectly represented, we recognize that family members’ views may not fully align with those of the residents themselves.

To address this feedback, the revised manuscript:

- Explicitly acknowledges that the exclusion of care home residents is a limitation of our study at the start of the manuscript
- Makes it clearer, on page 2 of the manuscript, that the primary aim of this study is to develop an information resource for health and social care professionals involved in care planning
- Provides a clear rationale for this decision on page 9 of manuscript
- Discuss how the involvement of family members partially addresses this gap, while noting the potential differences in perspectives.
- Notes the need for further work.

- Refers to an additional three studies, details below, which discuss the significant role that residents’ family and friend can play in supporting decisions related to their care.

1. Moermans VR, Mengelers AM, Bleijlevens MH, et al.; Caregiver decision-making concerning involuntary treatment in dementia care at home. Nursing Ethics 2022;29(2):330-343. doi: 10.1177/09697330211041742.
2. Omori M, Baker C, Jayasuriya J, et al.; Maintenance of Professional Boundaries and Family Involvement in Residential Aged Care. Qualitative Health Research 2019;29(11):1611-1622. doi: 10.1177/1049732319839363.
3. Bavelaar L, Visser M, Walshe C, et al.; The impact of the mySupport advance care planning intervention on family caregivers’ perceptions of decision-making and care for nursing home residents with dementia: pretest–posttest study in six countries. Age and Ageing 2023;52(3). doi: 10.1093/ageing/afad027.
